# Antibody–Drug Conjugates Beyond HER2 in Non-Small Cell Lung Cancer (NSCLC): Mechanisms, Emerging Targets, and Future Directions

**DOI:** 10.3390/biom16050677

**Published:** 2026-05-02

**Authors:** Ahmed Ismail, Aakash Desai, George R. Simon, Yanis Boumber

**Affiliations:** 1 Department of Medicine, Eastern Virginia Medical School at Old Dominion University, Norfolk, VA 23507, USA; 2 Division of Hematology and Oncology, Department of Medicine, O’Neal Comprehensive Cancer Center, University of Alabama at Birmingham, Birmingham, AL 35487, USA; aakashdesai@uabmc.edu; 3 Department of Medical Oncology, Ohio Health, Columbus, OH 43228, USA; george.simon@ohiohealth.com; 4 Department of Medical Oncology, Ohio Health, Delaware, OH 43015, USA

**Keywords:** non-small cell lung cancer (NSCLC), antibody–drug conjugates (ADCs), TROP2, HER3, MET, CEACAM5, B7-H3, Nectin-4, toxicity management, biomarkers

## Abstract

Antibody–drug conjugates (ADCs) are a rapidly evolving class of oncology therapeutics that enable precise delivery of potent cytotoxic agents to tumor cells while minimizing systemic toxicity. While HER2-targeted ADCs such as trastuzumab deruxtecan (T-DXd) in HER2-mutant, Datopotamab deruxtecan (Dato-Dxd) in EGFR-mutant, and telisotumumab vedotin (Teliso-V) in MET IHC 3+ expressing lung cancer have already established a clinical role in non-small cell lung cancer (NSCLC), multiple ADCs targeting alternative antigens, including additional TROP2 ADCs, HER3, MET, CEACAM5, B7-H3, Nectin-4, and others, are now in advanced clinical development. This review synthesizes the current evidence for non-HER2 ADCs in NSCLC, highlighting mechanisms of action, clinical efficacy, safety profiles, biomarker strategies, and emerging resistance mechanisms. Key safety concerns, including interstitial lung disease (ILD), ocular toxicity, and peripheral neuropathy, are emphasized alongside approaches for re-challenge following toxicity. We further discuss next-generation ADC platforms, including bispecific and conditionally activated constructs, as well as combination strategies with immunotherapy. Collectively, ADCs beyond HER2 are poised to reshape treatment paradigms in NSCLC, offering hope for patients with limited therapeutic options. This review identifies current gaps, highlights ongoing research priorities, and proposes practical considerations for integrating these therapies into clinical practice.

## 1. Introduction

Non-small cell lung cancer (NSCLC) remains the leading cause of cancer-related mortality worldwide, accounting for approximately 85% of all lung cancer cases [1,2]. Despite advances in targeted therapy and immunotherapy, the prognosis for patients with advanced or refractory NSCLC remains poor, particularly in those with rare molecular subtypes or after multiple lines of treatment [3]. Conventional chemotherapy offers limited survival benefit and is associated with systemic toxicities, underscoring the need for more precise and effective treatment modalities [3].

Antibody–drug conjugates (ADCs) represent a novel class of therapeutics that combine the specificity of monoclonal antibodies with the cytotoxic potency of small-molecule drugs [2]. By targeting tumor-specific antigens, ADCs deliver cytotoxic payloads directly to cancer cells, thereby reducing off-target effects and improving the therapeutic index [4]. The clinical success of ADCs in hematologic malignancies and breast cancer has catalyzed their rapid development in solid tumors, including NSCLC [5,6]. The first clinically relevant ADC for NSCLC was trastuzumab deruxtecan (T-Dxd), which was approved by the FDA for HER2-mutant advanced/metastatic NSCLC in August of 2022. HER2-directed ADCs, most notably trastuzumab deruxtecan (T-DXd), were the first to establish proof-of-concept of ADC activity in NSCLC, demonstrating meaningful response rates and durable clinical benefit in patients harboring HER2 mutations and amplifications [7]. These results have validated ADCs as a viable treatment strategy in lung cancer, spurring interest in alternative targets beyond HER2 [8,9]. In May of 2025, the Food and Drug Administration (FDA) approved telisotuzumab vedotin-tllv, a c-Met-directed antibody and microtubule inhibitor conjugate, for adults with locally advanced or metastatic, non-squamous non-small cell lung cancer (NSCLC) with high c-Met protein overexpression [≥50% of tumor cells with strong (3+) staining] who received prior systemic therapy. More recently, in June 2025, the FDA granted approval to datopotamab deruxtecan-dlnk for adults with locally advanced or metastatic epidermal growth factor receptor (EGFR)-mutated non-small cell lung cancer (NSCLC) who have received prior EGFR-directed therapy and platinum-based chemotherapy.

Emerging ADCs in NSCLC are being developed against a variety of tumor-associated antigens, including TROP2, HER3, and MET. Early-phase and pivotal studies indicate these agents can induce clinically significant responses even in heavily pretreated populations, broadening the therapeutic landscape [10,11,12,13]. Despite their promise, ADCs present unique challenges. Toxicities such as interstitial lung disease (ILD), ocular effects, myelosuppression, and neuropathy are observed across ADC platforms and can limit treatment duration or necessitate discontinuation [14]. Additionally, tumor heterogeneity, variability in antigen expression, and the emergence of resistance mechanisms can compromise efficacy [15]. Optimal patient selection, biomarker-guided strategies, and careful monitoring for toxicity are therefore essential.

To date, reviews have primarily focused on HER2-directed ADCs or on broader solid tumor applications [2,7], leaving a gap in the literature regarding non-HER2 ADCs in NSCLC. This review aims to synthesize the current evidence on non-HER2 ADCs, including their mechanisms of action, clinical efficacy, safety profiles, biomarker considerations, and strategies for overcoming resistance. Additionally, we discuss next-generation ADC platforms and highlight future directions in the evolving landscape of lung cancer therapeutics.

## 2. ADC Design, Mechanisms, and Class Toxicities

### 2.1. ADC Architecture and Design Principles

Antibody–drug conjugates (ADCs) consist of three essential components: a monoclonal antibody, a cytotoxic payload, and a chemical linker that connects them. Each element is critical for therapeutic efficacy and safety.

The monoclonal antibody confers specificity by recognizing tumor-associated antigens. IgG1 is the most commonly used backbone due to its favorable half-life and effector functions, though IgG2 and IgG4 constructs are also used depending on the desired immune engagement [2,4,7,14].

The cytotoxic payload is a highly potent small molecule, often 100–1000 times more cytotoxic than conventional chemotherapy, enabling effective tumor cell killing at low systemic exposure. Standard payload classes in NSCLC ADCs include topoisomerase I inhibitors (e.g., DXd), microtubule inhibitors (e.g., monomethyl auristatin E [MMAE], monomethyl auristatin F [MMAF], maytansinoid derivative 4 [DM4]), and DNA alkylators (e.g., pyrrolobenzodiazepine dimers [PBD]) [2,4,7,14].

The linker connects the antibody to the payload and controls the release kinetics. Linkers can be categorized into cleavable and non-cleavable. Tumor-specific enzymes or acidic lysosomal environments (e.g., DXd linkers) hydrolyze cleavable linkers. However, non-cleavable linkers rely on complete proteolytic degradation of the antibody for payload release, reducing off-target exposure [4,14]. In DXd-based ADCs such as datopotamab deruxtecan, the tetrapeptide linker is selectively cleaved by lysosomal proteases but can also be susceptible to limited systemic cleavage, releasing a highly membrane-permeable topoisomerase I inhibitor that readily diffuses into surrounding lung parenchyma and may contribute to the higher incidence of ILD seen in NSCLC cohorts [4,14]. In contrast, sacituzumab govitecan uses a hydrolyzable linker to SN-38, which is also membrane-permeable but displays a toxicity profile dominated by myelosuppression and gastrointestinal events, with less pronounced ILD signal in lung cancer studies, suggesting that linker stability, release kinetics, and payload physicochemical properties jointly shape organ-specific toxicity [4,14].

### 2.2. Mechanisms of Action

**Figure 1:**
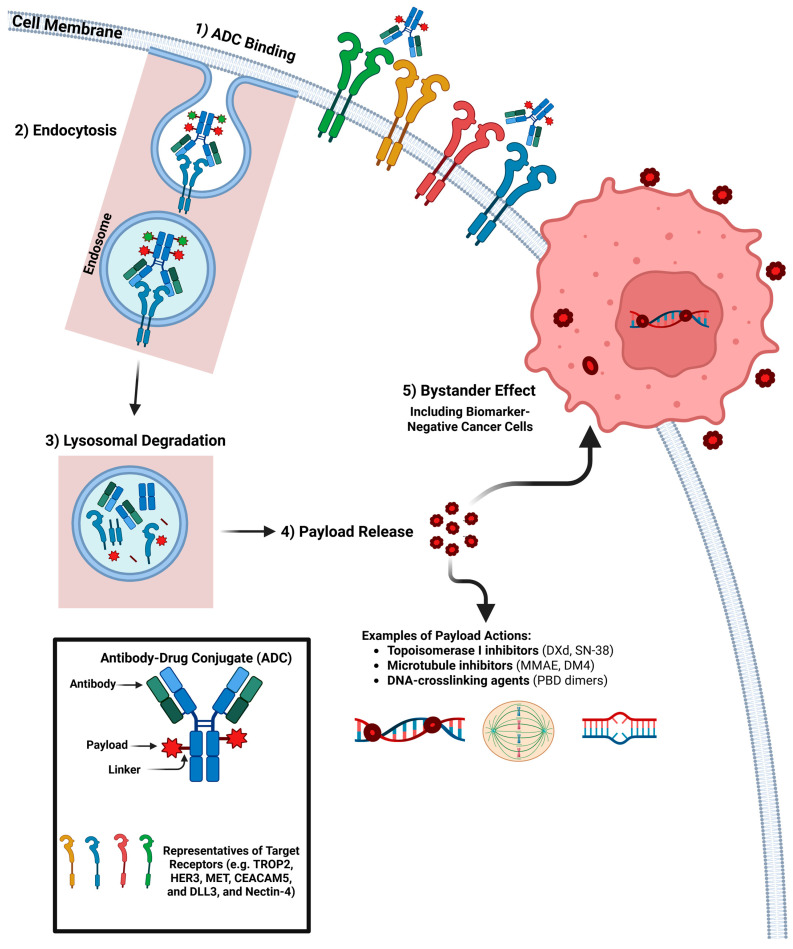
Non-HER2 Alterations and Mechanism of Action of Non-HER2 ADCs in NSCLC. ADCs bind tumor-associated antigens (e.g., TROP2, HER3, MET, CEACAM5), undergo receptor-mediated endocytosis, and release cytotoxic payloads following lysosomal degradation. Payloads such as DXd, SN-38, MMAE, and DM4 induce DNA damage or microtubule disruption, leading to tumor cell death. Membrane-permeable payloads further mediate a bystander effect, enabling killing of adjacent antigen-negative tumor cells and overcoming intratumoral heterogeneity.

ADCs can exert anti-tumor effects through multiple mechanisms. The ADC binds to the antigen on the tumor cell surface, undergoes endocytosis (targeted internalization), and releases the cytotoxic payload intracellularly [16] (Figure 1 and Table 1).

Another important mechanism is the bystander effect, where certain payloads, particularly membrane-permeable topoisomerase inhibitors (DXd), can diffuse into neighboring tumor cells, killing antigen-negative cells and addressing intratumoral heterogeneity [2,15,17]. However, this same membrane permeability that enables bystander killing also allows payload diffusion into non-malignant epithelial, endothelial, and immune cells within the lung microenvironment, which may contribute to ‘on-target/off-tumor’ and ‘off-target’ toxicities such as ILD, stomatitis, and myelosuppression observed with DXd- and SN-38-based ADCs. Thus, the bystander effect represents a therapeutic trade-off between improved coverage of heterogeneous tumors and increased risk of collateral injury to normal tissues [18].

Additionally, in immune engagement some ADCs retain Fc-mediated effector functions, recruiting immune cells for antibody-dependent cellular cytotoxicity (ADCC) or complement-mediated cytotoxicity (CDC); although, many designs minimize this to reduce toxicity [19].

**Table 1:** Design and Characteristics of Major Non-HER2 ADCs in NSCLC.

ADC	Target Antigen	Antibody Type	Payload	Payload Class	Linker Type	Bystander Effect	Key Clinical Study	DAR
Datopotamab deruxtecan (Dato-DXd)	TROP2	Humanized IgG1	DXd	Topoisomerase I inhibitor	Cleavable tetrapeptide linker	Yes (strong)	Phase III—TROPION-Lung01	~4
Sacituzumab govitecan (SG)	TROP2	Humanized IgG1	SN-38	Topoisomerase I inhibitor	Hydrolysable linker	Yes (strong)	Phase III—EVOKE-01	~7.6
Sacituzumab tirumotecan (MK-28701, SKB264)	TROP2	Humanized IgG1	Belotecan derivative	Topoisomerase I inhibitor	Cleavable linker	Yes (strong)	Phase III—OptiTROP-Lung04	-
Patritumab deruxtecan (HER3-DXd)	HER3 (ERBB3)	Fully human IgG1	DXd	Topoisomerase I inhibitor	Cleavable peptide linker	Yes (strong)	Phase II—HERTHENA-Lung01	~8
Izalontamab brengitecan (BL-B01D1)	EGFR/HER3	Bispecific antibody		Topoisomerase I inhibitor	Cleavable linker	Yes (strong)	Phase I—first-in-human study	-
Telisotuzumab vedotin (Teliso-V)	MET	Humanized IgG1	MMAE	Microtubule inhibitor	Valine-citrulline protease-cleavable linker	Limited	Phase II—LUMINOSITY	~3–4
Tusamitamab ravtansine (SAR408701)	CEACAM5	Humanized IgG1	DM4	Microtubule inhibitor	Cleavable disulfide linker	Limited	Phase I/II clinical studies	~3–4
Ifinatamab deruxtecan (DS-7300)	B7-H3 (CD276)	Humanized IgG1	DXd	Topoisomerase I inhibitor	Cleavable peptide linker	Yes (strong)	Phase I/II early clinical studies	-
Enfortumab vedotin	Nectin-4	Fully human IgG1	MMAE	Microtubule inhibitor	Protease-cleavable linker	Limited	Phase II—EV-202	~3–4

Table is adapted from contemporary ADC platform reviews, NSCLC-focused ADC overviews, and the primary development reports for each construct [4,10,11,14,17,20,21,22,23,24,25,26,27,28,29]. ADC: antibody–drug conjugate. B7-H3: B7 homolog 3 (CD276). CD276: cluster of differentiation 276. CEACAM5: carcinoembryonic antigen-related cell adhesion molecule 5. DAR: drug-to-antibody ratio. DM4: maytansinoid microtubule inhibitor. DXd: deruxtecan. EGFR: epidermal growth factor receptor. SG: sacituzumab govitecan. SN-38: active metabolite of irinotecan. HER3: human epidermal growth factor receptor 3. MET: mesenchymal–epithelial transition receptor. MMAE: monomethyl auristatin E. Nectin-4: nectin cell adhesion molecule 4. NSCLC: non-small cell lung cancer.

### 2.3. Key Toxicities Across ADC Platforms and Re-Challenge Considerations

ADCs exhibit a unique toxicity profile influenced by the antibody target, payload, and linker properties. Understanding these toxicities is essential for clinical management in NSCLC. Rechallenge after toxicity is feasible in select patients, particularly if the adverse event resolves to Grade ≤ 1 (Table 2).

ILD/Pneumonitis is most frequent with DXd-based ADCs (T-DXd, Dato-DXd, HER3-DXd), ranging from 5–15% across trials, with most events grade 1–2 (~77%) and ~2.2% fatal (grade 5) [30]. It usually presents with cough, dyspnea, hypoxemia, and ground-glass opacities on CT [31]. Its management and prompt recognition are critical. Grade ≥2 ILD may require ADC discontinuation and corticosteroid therapy. Mild cases (grade 1) may be monitored with dose interruption and gradual re-challenge after resolution [31,32]. Risk factors usually include pre-existing lung disease, prior thoracic radiation, older age, and high cumulative dose [30].

Ocular toxicity is typically observed with DM4 payloads (e.g., Tusamitamab Ravtansine). It manifests as keratitis, blurred vision, photophobia, or microcystic corneal changes. Prevention and management mainly rely on prophylactic preservative-free lubricating eye drops, ophthalmology consultations, and dose modifications as needed [33].

Peripheral neuropathy is observed with MMAE/MMAF payloads (Telisotuzumab Vedotin, other microtubule inhibitors). Presentation typically includes paresthesia, numbness, and tingling in the extremities, which can be cumulative. Management typically involves dose interruption, dose reduction, and symptomatic therapy (e.g., gabapentinoids). It is usually reversible, but chronic neuropathy may occur in a minority of patients [34].

Myelosuppression and cytopenias are observed with DXd payloads (HER3-DXd, Dato-DXd) and SN-38 (SG). It manifests as neutropenia and anemia. Thrombocytopenia may also occur, particularly with DXd- or SG-based ADCs. Neutropenic fever is uncommon. Dose modification, growth factor support, and transfusions may be necessary [34].

Gastrointestinal toxicity is particularly observed with topoisomerase I-based ADCs. Nausea, vomiting, and diarrhea are usually the most common manifestations. Stomatitis is also observed with DXd payloads. Toxicity is generally manageable with supportive care (antiemetics, hydration, and oral care) [35,36].

Taken together, available data suggest that ILD is most tightly linked to DXd-based constructs, whereas SN-38-conjugated ADCs primarily cause neutropenia and diarrhea; this divergence likely reflects differences in linker stability, systemic deconjugation, and payload distribution rather than target antigen alone. Furthermore, these clinical patterns support a model in which systemically released, membrane-permeable topoisomerase I payloads can reach non-tumor lung tissue and mucosal surfaces, particularly in heavily pretreated NSCLC patients with fragile pulmonary reserve, underscoring the need for early recognition and conservative dose modification.

Mild cases of toxicities, especially grade 1, may be monitored with dose interruption and gradual re-challenge after resolution. However, careful multidisciplinary review is usually required for grade 2 events. Re-challenge is generally discouraged after grade ≥3 events. Limited published data suggest that re-challenge can achieve tumor response while maintaining tolerability, but careful monitoring is mandatory [31,32].

## 3. Non-HER2 ADCs in NSCLC

Non-HER2 antibody–drug conjugates (ADCs) are rapidly expanding the therapeutic landscape of non-small cell lung cancer (NSCLC), targeting a diverse set of tumor-associated antigens beyond HER2 and demonstrating encouraging clinical activity across multiple molecular and histologic subtypes (Table 3).

### 3.1. TROP2-Targeted ADCs

Trophoblast cell surface antigen 2 (TROP2) is a transmembrane glycoprotein highly expressed in many epithelial malignancies, including NSCLC. Its expression is generally low in normal tissues, making it an attractive ADC target [46]. TROP2 expression is observed in approximately 70–90% of NSCLC specimens [46]. Preclinical studies demonstrate that TROP2 overexpression promotes tumor proliferation, survival, and metastasis, providing a rationale for targeted therapy [47].

#### 3.1.1. Datopotamab Deruxtecan (Dato-DXd)

Dato-DXd is an IgG1 monoclonal antibody targeting TROP2, conjugated via a cleavable linker to a DXd payload (a topoisomerase I inhibitor). It is designed to exploit both targeted cytotoxicity and bystander killing of adjacent antigen-negative tumor cells [37].

TROPION-Lung01 (Phase III) [38] comprehensively assesses Dato-DXd. The population in this study consists of patients with previously treated advanced NSCLC (squamous and non-squamous), who received platinum-based chemotherapy ± an immune checkpoint inhibitor. The study included 299 and 305 patients who were randomly assigned to receive Dato-DXd or docetaxel, respectively. The median progression-free-survival (mPFS) was 4.4 months with Dato-DXd compared to 3.7 months with docetaxel, with a hazard ratio [HR] of 0.75 (*p* = 0.004). The median overall survival (mOS) was 12.9 months and 11.8 months, respectively, with an HR of 0.94 (*p* = 0.53). The confirmed objective response by blinded independent central review was 26.4% (95% CI, 21.5 to 31.8) with Dato-DXd and 12.8% (95% CI, 9.3 to 17.1) with docetaxel.

In the subgroup analysis for non-squamous NSCLC [38], the median PFS was 5.5 versus 3.6 months, with an HR of 0.63. The mOS was 14.6 versus 12.3 months, with a statistically insignificant HR of 0.84. In the squamous histology subgroup [38], the mPFS was 2.8 versus 3.9 months, with a statistically insignificant HR of 1.41. The mOS was 7.6 versus 9.4 months, with a statistically insignificant HR of 1.32. This showed superior secondary efficacy outcomes for Dato-DXd compared with docetaxel in the non-squamous NSCLC subgroup. Grade ≥3 treatment-related adverse events (TRAEs) occurred in 25.6% and 42.1% of patients, in the Dato-DXd and docetaxel groups, respectively. Any-grade adjudicated drug-related pneumonitis/interstitial lung disease occurred in 8.8% and 4.1% of patients, respectively [38].

Although Dato-DXd is directed at TROP2, the pivotal TROPION-Lung01 Phase III publication does not include a prespecified analysis stratifying efficacy by baseline TROP2 expression level. However, a retrospective exploratory analysis using a novel quantitative computational pathology-based TROP2 scoring (TROP2-QCS, “normalized membrane ratio”) evaluated 352 biomarker-evaluable tumors from TROPION-Lung01 [39]. In the TROP2-QCS–positive subgroup (*n* = 107), Dato-DXd achieved an mPFS of 6.9 months, objective response rate (ORR) of 32.7%, and maintained a favorable HR for progression or death compared to docetaxel. By contrast, in QCS-negative tumors, mPFS dropped to 2.9 months, and ORR was 16.9%. These findings suggest that while the original Phase III data do not permit robust conclusions about TROP2 expression–response relationships, retrospective quantitative biomarker analysis indicates that higher TROP2 expression (per QCS) may be associated with greater benefit. Together, this underscores the importance of prospective biomarker-guided stratification in future Dato-DXd trials. These findings, together with the lack of a clear predictive role for TROP2 by conventional IHC in sacituzumab govitecan studies, illustrate that binary ‘high vs. low’ antigen thresholds may inadequately capture ADC sensitivity and contribute to the apparent biomarker paradox observed in NSCLC.

Although Dato-DXd has demonstrated clinical activity in previously treated NSCLC, additional evidence supporting its use in biomarker-defined populations comes from the phase II TROPION-Lung05 study [10]. This trial evaluated patients with advanced or metastatic NSCLC harboring actionable genomic alterations who had progressed after prior targeted therapy and platinum-based chemotherapy. In this molecularly selected population, Dato-DXd demonstrated clinically meaningful antitumor activity with a confirmed ORR of 43.6% (95% CI, 33.7–53.9) and a mDoR of approximately 6.5 months, with responses observed across multiple oncogenic subgroups, including EGFR-mutated tumors [10]. The safety profile was generally manageable and consistent with prior studies of DXd-based ADCs, with the most common TRAEs including stomatitis, nausea, and fatigue, while adjudicated ILD occurred in a small proportion of patients. These findings are particularly relevant because the subsequent U.S. FDA accelerated approval of Dato-DXd for previously treated EGFR-mutated NSCLC was primarily supported by data from TROPION-Lung05, with additional supportive evidence from the phase III TROPION-Lung01 trial. Together, these results suggest that the most robust current clinical evidence for Dato-DXd in NSCLC lies within genomically selected populations, highlighting the importance of biomarker-guided patient selection when integrating ADC therapies into the treatment paradigm of NSCLC.

Since 2025, datopotamab deruxtecan has been approved for patients with locally advanced or metastatic epidermal growth factor receptor (EGFR)-mutated non-small cell lung cancer (NSCLC) who have received prior EGFR-directed therapy and platinum-based chemotherapy. This FDA approval was primarily based on the phase II TROPION-Lung05 study, which demonstrated a confirmed objective response rate of 43.6% and a median duration of response of approximately 6.5 months, with additional supportive evidence from the phase III TROPION-Lung01 trial [10,38].

#### 3.1.2. Sacituzumab Govitecan (SG; IMMU-132)

SG is a humanized IgG1 monoclonal antibody targeting TROP2, conjugated via a hydrolysable linker to SN-38, the active metabolite of irinotecan. By combining targeted antibody delivery with a potent topoisomerase I inhibitor payload, SG is designed to selectively deliver cytotoxic therapy to TROP2-expressing tumor cells while limiting systemic exposure. The hydrolysable linker allows controlled release of SN-38 in the tumor microenvironment, which may also contribute to a bystander effect, enabling killing of adjacent antigen-low or -negative tumor cells [48].

In a single-arm multicenter trial [40], including 54 patients, SG was evaluated in patients with heavily pretreated metastatic NSCLC, including both squamous and non-squamous histologies. In the intention-to-treat population, ORR was approximately 17%, mPFS was 5.2 months, and OS was 9.5 months, demonstrating clinically meaningful activity in a population with limited treatment options. Grade 3 or higher TEAEs included neutropenia (28%), diarrhea (7%), nausea (7%), fatigue (6%), and febrile neutropenia (4%). These toxicities were generally manageable with supportive care measures, dose modifications, or growth factor support, consistent with the known safety profile of SN-38-based ADCs. Notably, more than 90% of 26 assessable archival tumor specimens were highly positive (2+, 3+) for Trop-2 by immunohistochemistry, which suggests that Trop-2 is not a predictive biomarker for response.

Although SG was initially evaluated in early-phase and basket studies across mixed tumor histologies, a dedicated Phase III trial in NSCLC (EVOKE 01, NCT05089734) [41] has recently been completed. The trial did not meet its primary endpoint of overall survival in the overall population, though some subgroups showed numerical improvements in survival outcomes. These results underscore that confirmatory evidence of clinical benefit over standard chemotherapy remains limited, particularly in the broader NSCLC population. Despite this, SG continues to represent a salvage therapy option for patients with TROP2-positive NSCLC who have progressed on prior platinum-based chemotherapy or immunotherapy. Preclinical studies further suggest potential synergy when SG is combined with other targeted therapies or immune checkpoint inhibitors, which may inform future combination strategies and clinical trial design [49,50].

Although the phase III EVOKE-01 trial did not demonstrate an improvement in overall survival with SG in the overall population of previously treated NSCLC, ongoing studies are investigating whether this agent may have greater therapeutic impact in earlier lines of therapy, particularly in combination with immunotherapy. The phase II EVOKE-02 study evaluates SG in combination with pembrolizumab, with or without platinum-based chemotherapy, in patients with advanced or metastatic NSCLC without actionable genomic alterations [51]. In parallel, the ongoing phase III EVOKE-03 trial is comparing SG plus pembrolizumab with pembrolizumab monotherapy as first-line treatment for metastatic NSCLC with PD-L1 tumor proportion score ≥50% [52]. These trials aim to determine whether incorporating sacituzumab govitecan into immunotherapy-based frontline regimens may enhance antitumor activity and improve clinical outcomes compared with immune checkpoint inhibitor therapy alone. Collectively, these studies may clarify whether sacituzumab govitecan is better positioned as part of combination strategies in earlier treatment settings, rather than as single-agent salvage therapy in heavily pretreated NSCLC populations.

#### 3.1.3. Sacituzumab Tirumotecan (MK-28701, SKB264)

Sacituzumab Tirumotecan, licensed by Merck from Kelun Biotech, is a next-generation TROP2-directed antibody–drug conjugate composed of a humanized anti-TROP2 IgG1 monoclonal antibody conjugated via a cleavable linker to a topoisomerase I inhibitor payload (belotecan-derivative), designed to enhance tumor-selective delivery and maintain a bystander effect similar to other SN-38-based ADCs. This construct is designed to enhance tumor-selective delivery, improve payload stability, and maintain a bystander effect similar to other membrane-permeable topoisomerase I-based ADCs. Compared with earlier TROP2-directed ADCs, sacituzumab tirumotecan incorporates optimized linker–payload chemistry that may improve pharmacokinetics and therapeutic index, potentially contributing to enhanced antitumor activity [20].

In the phase 3 randomized OptiTROP-Lung04 trial, sacituzumab tirumotecan demonstrated a significant improvement in PFS compared with platinum-based doublet chemotherapy in patients with EGFR-mutant NSCLC who had progressed on prior EGFR TKI therapy. The mPFS was of 8.3 versus 4.3 months, respectively. Additionally, the 18-month OS was 65.8% versus 48% in favor of sacituzumab tirumotecan, supporting a clinically meaningful survival benefit in this population. Notably, the magnitude of benefit observed in this molecularly defined subgroup appears greater than that reported with earlier TROP2 ADCs in unselected NSCLC populations, highlighting the importance of biomarker-driven patient selection [42].

#### 3.1.4. Safety Considerations for TROP2 ADCs

ILD/Pneumonitis is rare but has been observed with DXd-based payloads (Dato-DXd). Myelosuppression is notable for SG due to SN-38 payload, and neutropenia may require G-CSF support. Gastrointestinal toxicity, especially diarrhea and nausea, is common. Dose modification is often required in such cases. Stomatitis is reported with Dato-DXd, and prophylactic oral care is recommended [37,38,39,40,41,48,49,50]. The safety profile of sacituzumab tirumotecan was generally consistent with topoisomerase I-based ADCs, with manageable hematologic and gastrointestinal toxicities, including neutropenia and diarrhea, although the incidence and severity of adverse events may differ from SG due to differences in payload and linker design [42].

#### 3.1.5. Rechallenge Considerations

Historically, limited data suggest that rechallenge with ADCs may be feasible in selected patients only after careful multidisciplinary review and resolution of lower-grade toxicity, but rechallenge after higher-grade (≥Grade 2) events is generally discouraged. Pooled analyses and case reports in the T-DxD experience indicate that retreatment following resolution of Grade 1 ILD has been performed safely in selected patients and can prolong treatment in some cases; however, regulatory product information and toxicity management guidance recommend permanent discontinuation for Grade ≥2 ILD except in exceptional circumstances. A few single-patient case reports describe successful rechallenge after Grade 2 ILD with close monitoring and steroid management, but these are anecdotal and do not change guideline recommendations. For ADC-related ILD, current product labels and expert consensus recommend permanent discontinuation of the ADC for Grade ≥2 events, given the risk of recurrent and potentially fatal (Grade 5) pneumonitis. Rechallenge after Grade 2 or higher ILD is generally contraindicated in routine practice and should be considered only under exceptional circumstances, ideally within a clinical trial, with multidisciplinary input, explicit patient consent, and intensive radiologic and clinical monitoring. Only in the setting of Grade 1 ILD that fully resolves with prompt interruption and corticosteroids is cautious rechallenge sometimes considered, and even then, the risk–benefit ratio must be carefully individualized [30,53,54].

### 3.2. MET-Targeted ADCs

MET is a receptor tyrosine kinase that plays a central role in oncogenic signaling pathways regulating tumor cell proliferation, survival, motility, and metastatic dissemination. Aberrant MET activation can occur through several mechanisms, most notably MET exon 14 (METex14) skipping mutations, gene amplification, and protein overexpression [55]. METex14-skipping alterations are identified in approximately 3–5% of NSCLC cases and are strongly associated with aggressive tumor biology and inferior clinical outcomes [55]. This is in part due to impaired ubiquitination and degradation of the MET receptor, leading to sustained downstream signaling. MET protein overexpression, which may arise independently of METex14 mutations or amplification, is even more common and contributes to resistance against multiple targeted therapies [56]. MET is also implicated as one of the key mechanisms of TKI resistance in EGFR mutant NSCLC after progression of EGFR TKIs [57]. Additionally, MET overexpression is observed in approximately 25–75% of NSCLC cases, while MET amplification occurs in ~5–20% of tumors and is enriched following EGFR TKI progression [56]. It is therefore a valid target for ADC development in NSCLC, regardless of genetic driver mutation subtypes.

Given the biological dependence of these tumors on MET signaling, MET-directed ADCs have emerged as a promising therapeutic strategy. These ADCs combine selective targeting of MET-overexpressing tumor cells with potent cytotoxic payload delivery, offering a potential advantage in tumors that have developed resistance to MET tyrosine kinase inhibitors (TKIs), through bypass pathway activation, incomplete inhibition, or intratumoral heterogeneity [56]. Early preclinical and clinical studies suggest that MET-targeted ADCs can retain activity even in the setting of TKI resistance, making them particularly attractive for patients with MET-driven NSCLC who have progressed on standard targeted therapies [56].

#### Telisotuzumab Vedotin (Teliso-V)

Teliso-V is a fully humanized IgG1 monoclonal antibody engineered to selectively recognize the extracellular domain of the MET receptor. The antibody is conjugated, via a protease-cleavable valine-citrulline linker, to monomethyl auristatin E (MMAE). MMAE is a highly potent microtubule-disrupting cytotoxic agent that binds tubulin, inhibiting microtubule polymerization and inducing mitotic arrest and apoptosis [21,58]. Preclinical studies demonstrated potent antitumor activity in MET-overexpressing tumor models, and subsequent phase I/II clinical evaluation confirmed the feasibility of this strategy, showing encouraging responses, particularly in MET-high NSCLC [21,58].

The LUMINOSITY Trial [22] enrolled 172 patients with non-squamous, EGFR wild-type NSCLC whose tumors overexpressed c-Met (Immunohistochemistry (IHC)-defined). Eligible patients had locally advanced/metastatic Met-overexpressing NSCLC and ≤2 previous lines of therapy, including ≤1 line of systemic chemotherapy. Teliso-V dosage was 1.9 mg/kg once every 2 weeks. The reported data included a full-cohort ORR of 28.6%, mDoR of 8.3 months, mOS of 14.5 months, and mPFS of 5.7 months. In the subsequent analysis for MET-high and intermediate, ORR was 34.6% and 22.9%, respectively [22]. Additionally, the mDoR was 9.0 and 7.2 months, mOS was 14.6 and 14.2 months, and mPFS was 5.5 and 6.0 months in the MET-high and intermediate groups, respectively [22]. The most common any-grade TRAEs were peripheral sensory neuropathy (30%), peripheral edema (16%), and fatigue (14%), with peripheral sensory neuropathy being the most common reported grade ≥3 TRAEs (7%) [22].

In 2025, FDA approved Teliso-V for patients with locally advanced or metastatic, non-squamous non-small cell lung cancer (NSCLC) with high c-Met protein overexpression [≥50% of tumor cells with strong (3+) staining] who received prior systemic therapy.

Such results show that MET-targeted ADCs can offer a therapeutic option for MET-overexpressing NSCLC, particularly for high MET-expressing patients (as measured by IHC), and likely also for patients ineligible for MET TKI therapy or with TKI resistance. Biomarker-driven selection is critical; efficacy is closely linked to MET expression levels. Combination with immunotherapy or other targeted agents is under investigation in early-phase trials [58,59,60].

### 3.3. HER3-Targeted ADCs, Including Bispecifics

HER3 (ErbB3) is a member of the EGFR family and is widely expressed in NSCLC, particularly in EGFR-mutated tumors. HER3 lacks strong kinase activity but heterodimerizes with EGFR and HER2, promoting PI3K/AKT signaling and resistance to EGFR TKIs [2]. HER3 expression is nearly universal in EGFR-mutant NSCLC, making it a compelling ADC target, especially in the context of TKI resistance [61,62].

#### 3.3.1. Patritumab Deruxtecan (HER3-DXd)

HER3-DXd is composed of a humanized IgG1 monoclonal antibody specifically directed against the human epidermal growth factor receptor 3 (HER3), which is conjugated via a cleavable peptide-based linker to a highly potent topoisomerase I inhibitor payload, DXd [11]. Selective binding to HER3-expressing tumor cells allows internalization and intracellular release of payload, thereby inducing apoptosis in targeted cells. Notably, the membrane-permeable DXd induces a bystander killing effect, eliminating neighboring tumor cells with low or heterogeneous HER3 expression [11]. This property is particularly advantageous in tumors with intratumoral HER3 heterogeneity, such as EGFR-mutant NSCLC, where some tumor cell populations may express lower levels of HER3 [11].

The HERTHENA-Lung01, phase II study, assessed the effectiveness of HER3-DXd in NSCLC [11]. The study included patients with advanced EGFR-mutated NSCLC who had previously received EGFR TKI therapy and platinum-based chemotherapy (PBC). In total, 225 patients received HER3-DXd 5.6 mg/kg once every 3 weeks. The reported ORR was 29.8%, the median duration of response (mDoR) was 6.4 months, the mPFS was 5.5 months, and the mOS was 11.9 months.

The subgroup of patients who had previously received osimertinib and PBC demonstrated comparable clinical outcomes to the overall population. Therapeutic activity was evident across tumors with varying levels of HER3 membrane expression, as well as across different mechanisms of resistance to EGFR TKIs. Among patients presenting with untreated brain metastases at baseline (*n* = 30), the confirmed intracranial ORR was 33.3%. Overall, the safety profile remained consistent with prior studies and was generally manageable and well tolerated.

HER3-DXd demonstrated a manageable safety profile consistent with the known effects of DXd-based ADCs [11]. Adjudicated ILD occurred in 5.3% of patients, underscoring the need for careful monitoring, although most events were low grade and manageable with corticosteroid therapy. Other commonly reported treatment-emergent adverse events (TEAEs) included nausea, stomatitis, fatigue, and gastrointestinal toxicities, predominantly of mild to moderate severity. Hematologic toxicities were also observed, with grade ≥3 neutropenia and thrombocytopenia reported in approximately 19% and 21% of patients, respectively [11]. The frequency and severity of these events were generally consistent with previous early-phase and preclinical studies [63,64], and most adverse events could be mitigated with dose modification, supportive care, or temporary treatment interruption. Collectively, these findings indicate that HER3-DXd has a tolerable safety profile while delivering clinically meaningful antitumor activity in patients with EGFR-mutant NSCLC.

Beyond the phase II HERTHENA-Lung01 results, the clinical development of HER3-DXd has progressed to confirmatory phase III evaluation in the HERTHENA-Lung02 trial. This global randomized study compares HER3-DXd with platinum-based chemotherapy in patients with locally advanced or metastatic EGFR-mutated non-squamous NSCLC who have previously received EGFR TKI therapy, including progression after a third-generation EGFR TKI [65]. In September 2024, the sponsor announced that HERTHENA-Lung02 met its primary endpoint of PFS compared with platinum-based chemotherapy, with a safety profile consistent with prior studies of HER3-DXd and no new safety signals identified at the time of analysis [66]. Unfortunately, in May 2025, Daiichi Sankyo and Merck withdrew the biologics license application for Patritumab Deruxtecan and halted clinical development after the HERTHENA-Lung02 trial did not meet the OS primary endpoint.

#### 3.3.2. Izalontamab Brengitecan (Iza-Bren, BL-B01D1)

Iza-Bren is a novel bispecific antibody–drug conjugate targeting both EGFR and HER3, designed to overcome tumor heterogeneity and resistance mechanisms that limit the efficacy of single-target ADCs. By simultaneously engaging EGFR and HER3, this dual-targeting strategy enhances tumor selectivity and internalization, while potentially addressing bypass signaling pathways that contribute to resistance following EGFR TKI therapy. The antibody is conjugated via a cleavable linker to a potent topoisomerase I inhibitor payload, enabling intracellular release of cytotoxic drug and facilitating a bystander effect in adjacent tumor cells with variable antigen expression [23].

Early-phase clinical studies (phase Ia/Ib and phase II multicohort trials) have demonstrated encouraging antitumor activity of Iza-Bren in patients with advanced EGFR-mutant NSCLC who have progressed on third-generation EGFR TKIs, including osimertinib. In these heavily pretreated populations, the agent has shown pooled confirmed ORR (~44–50%) and mDoR (~6–8 months) across diverse resistance mechanisms, including both on-target and bypass alterations. Additionally, activity has been observed irrespective of HER3 expression levels, supporting the rationale for dual EGFR/HER3 targeting in overcoming intratumoral heterogeneity [23]. However, these estimates come from relatively small cohorts with limited follow-up and should be considered hypothesis-generating; they are not directly comparable to mature phase III data from studies such as TROPION-Lung01 of Dato-DXd.

The safety profile of izalontamab brengitecan appears consistent with other topoisomerase I-based ADCs, with manageable hematologic and gastrointestinal toxicities, including neutropenia, nausea, and diarrhea. As with other ADCs in this class, careful monitoring for overlapping toxicities remains important, particularly in patients previously exposed to multiple lines of therapy.

### 3.4. CEACAM5-Targeted ADCs

Carcinoembryonic antigen-related cell adhesion molecule 5 (CEACAM5, also known as CEA) is a glycoprotein expressed in a subset of NSCLC, particularly adenocarcinomas. CEACAM5 is minimally expressed in normal lung tissue, enabling selective tumor targeting [67]. Expression levels are highly heterogeneous, making patient selection critical.

#### Tusamitamab Ravtansine (SAR408701)

Tusamitamab Ravtansine is an anti-CEACAM5 IgG1 monoclonal antibody conjugated to DM4 (microtubule inhibitor) via a cleavable disulfide linker. The conjugate is efficiently internalized upon binding to CEACAM5, leading to intracellular DM4 release and microtubule disruption in CEACAM5-expressing tumor cells. Early-phase clinical studies have demonstrated antitumor activity in CEACAM5-high NSCLC, particularly adenocarcinoma, supporting further investigation in biomarker-selected populations [24].

A Phase 1b dose-expansion study of Tusamitamab Ravtansine (SAR408701) in non-squamous NSCLC with CEACAM5 expression (high or moderate) reported safety and preliminary antitumor activity [25]. In that study, confirmed ORR was 20.3% in the high-CEACAM5 expression cohort and 7.1% in the moderate-CEACAM5 expression cohort, when defined by IHC cutoffs [25]. In addition, the mDoR was 6.7 months in the high-CEACAM5 expression group. The TRAEs occurred in 78.3% of patients, with 37.0% requiring dose modifications, and 5.4% requiring discontinuation of treatment [25]. The most common TRAEs included asthenia (37.0%) and dyspnea (23.9%). Corneal adverse events occurred in 38.0%, which were typically grade 1/2, reversible, and manageable by dose modifications [25]. However, despite these early signals of activity, subsequent phase III evaluation did not confirm clinical benefit. The randomized CARMEN-LC03 trial failed to meet its dual primary endpoint of improving progression-free survival compared with standard therapy, and the sponsor subsequently announced discontinuation of the clinical development program for tusamitamab ravtansine in lung cancer [43]. Despite stringent CEACAM5 IHC selection, tusamitamab ravtansine did not improve PFS or OS versus docetaxel in CARMEN-LC03, underscoring that high target expression alone does not guarantee clinical benefit and that other factors, payload class, off-tumor expression, and microtubule resistance, may negate the theoretical advantage of biomarker enrichment. These results highlight the challenges of translating early-phase ADC activity into definitive clinical benefit in biomarker-selected NSCLC populations.

### 3.5. Other Emerging ADC Targets in NSCLC

#### 3.5.1. Nectin-4

Nectin-4 (poliovirus receptor-related protein 4, PVRL4) is a calcium-independent immunoglobulin-like cell adhesion molecule belonging to the nectin family, involved in cell–cell junction formation and epithelial tissue organization. It is expressed in a subset of NSCLC, though its levels and prevalence vary across histologic subtypes. Preclinical studies have shown that Nectin-4 ADCs can induce potent antitumor effects in Nectin-4-positive NSCLC models, supporting its potential as a therapeutic target. Importantly, Nectin-4 expression is typically low in most normal adult tissues, which may reduce off-target toxicity when using Nectin-4-directed therapies. Ongoing investigations with Nectin-4 ADCs thus represent a promising translational avenue for NSCLC, especially in tumors without actionable driver mutations [26]. Clinical data for Nectin-4-targeted ADCs in NSCLC remain limited but available. In the phase II EV-202 study of enfortumab vedotin in previously treated NSCLC, the non-squamous cohort demonstrated a confirmed ORR of 14%, with a mPFS of 4.1 months and mOS of 10.5 months [27]. Although predefined efficacy thresholds were not met, these findings provide early clinical proof-of-concept for Nectin-4-directed therapy in NSCLC, supporting continued development with improved patient selection strategies.

#### 3.5.2. B7-H3

B7-H3 is a type I transmembrane immunoregulatory protein belonging to the B7 family of immune checkpoint molecules, involved in modulation of T-cell-mediated immune responses. It is frequently overexpressed in NSCLC, with immune-histochemical studies demonstrating protein expression in up to 80% of tumors [68]. Overexpression of B7-H3 in NSCLC has been associated with more aggressive clinic-pathologic features, including nodal metastasis, poor differentiation, and shorter overall survival. This suggests that B7-H3 contributes to tumor progression beyond immune modulation [69]. As a result, B7-H3 has emerged as an attractive target for therapeutic development. Several preclinical studies have already engineered B7-H3-directed ADCs (and other modalities) that effectively kill B7-H3–positive NSCLC cell lines and xenografts, demonstrating proof-of-concept for CD276-directed therapy in lung cancer [70]. Published clinical efficacy data for B7-H3-targeted ADCs in NSCLC remain preliminary. In the phase I/II study of ifinatamab deruxtecan, a small squamous NSCLC subgroup demonstrated a confirmed ORR of 40% (2/5) and a median DoR of 4.3 months. However, cohort size was very limited and mature NSCLC-specific PFS and OS data have not yet been clearly reported [28,29].

In summary, emerging targets expand the ADC landscape beyond traditional antigens. Most early-phase studies emphasize proof-of-concept, tolerability, and biomarker-guided patient selection. Future studies are needed to validate clinical efficacy and establish optimal dosing.

## 4. Non-HER2 Biomarkers and Resistance Mechanisms

The efficacy of ADCs in NSCLC is usually strongly dependent on tumor surface antigen expression (Table 4). Therefore, biomarker-driven selection is essential to optimize response and minimize toxicity. IHC-Based Selection: TROP2, MET, CEACAM5, and HER3 expression is commonly assessed by IHC. Expression thresholds (e.g., 1+, 2+, 3+) are used to determine eligibility in most clinical trials. High expression generally correlates with higher response rates, though some ADCs (e.g., Dato-DXd) show activity across lower-expression tumors due to the bystander effect [38]. Genomic/Mutation-Based Selection: HER3-DXd efficacy is enriched in EGFR-mutated NSCLC, particularly after TKI resistance [44]. MET exon 14 skipping mutations or MET amplification can influence ADC sensitivity [71].

Heterogeneity Considerations: Tumor antigen expression can vary spatially (between primary and metastatic sites) and temporally (before vs. after treatment). This variability can lead to differential ADC efficacy across tumor sites and may contribute to the emergence of resistant clones. Serial biopsy or liquid biopsy approaches can help capture this dynamic expression and guide therapeutic decisions [72].

Biomarker paradox in ADCs: Collectively, these experiences highlight a biomarker paradox in ADC development, wherein high IHC expression (e.g., MET or CEACAM5) enriches for response in some settings yet fails to translate into survival benefit in others, while agents such as Dato-DXd show meaningful activity in unselected or low-expression tumors due to bystander killing. This paradox likely reflects intratumoral and inter-lesional heterogeneity, variability in IHC scoring across platforms, dynamic changes in antigen expression after prior therapies, and the dominant influence of payload-related pharmacology and resistance mechanisms.

**Table 4:** Biomarkers and Resistance Mechanisms associated with Non-HER2 ADCs in NSCLC.

ADC Target	Biomarker	Assessment Method	Biomarker Limitations	Major Resistance Mechanisms	Potential Strategies
TROP2 ADCs	TROP2 expression	IHC	High expression common but not predictive of response	Antigen loss, ABC transporter-mediated drug efflux, altered internalization, TOP1 mutations	Alternative ADC targets, ADC + ICI combinations, payload switching
HER3 ADCs	HER3 expression, EGFR mutation	IHC, genomic testing	Activity observed across HER3 expression levels; not strictly expression-dependent	Antigen heterogeneity, bypass signaling (PI3K/AKT), impaired internalization	Combination with EGFR TKIs, dual-target ADCs
Dual-target ADCs (EGFR/HER3 bispecifics)	EGFR mutation, HER3 expression, resistance alterations after EGFR TKI therapy	IHC, genomic testing	Early clinical validation; predictive value of HER3 expression remains uncertain	On-target EGFR resistance, bypass pathway activation, and antigen heterogeneity	Biomarker-selected post-TKI trials, dual-target ADC development, combination with EGFR TKIs
MET ADCs	MET overexpression	IHC	IHC variability and lack of standardized thresholds	Antigen variability, MET pathway reactivation, and downstream resistance pathways	Combination with MET TKIs, biomarker refinement
CEACAM5 ADCs	CEACAM5 expression	IHC	Heterogeneous expression; threshold-dependent efficacy	Antigen loss, microtubule resistance (tubulin alterations), drug efflux	Biomarker-guided selection, alternative payload ADCs
Emerging targets (Nectin-4)	Nectin-4 expression	IHC (exploratory)	Limited validation as predictive biomarkers	Early/unknown; likely antigen heterogeneity and payload resistance	Biomarker-driven trials, improved patient selection

Table is synthesized from target-specific clinical trials and ADC resistance/biomarker reviews [11,22,23,24,26,27,37,44,46,56,67,73,74,75]. ABC: ATP-binding cassette. ADC: antibody–drug conjugate. AKT: protein kinase B. CEACAM5: carcinoembryonic antigen-related cell adhesion molecule 5. EGFR: epidermal growth factor receptor. HER3: human epidermal growth factor receptor 3. ICI: immune checkpoint inhibitor. IHC: immunohistochemistry. MET: mesenchymal–epithelial transition receptor. NSCLC: non-small cell lung cancer. PI3K: phosphoinositide 3-kinase. TOP1: topoisomerase I. TKI: tyrosine kinase inhibitor. TROP2: trophoblast cell surface antigen 2.

Despite promising initial activity, resistance to ADCs can develop through multiple mechanisms [15]. Antigen loss or downregulation in tumor cells may reduce surface expression of the target antigen, thereby preventing ADC binding, as observed in preclinical models of HER3 and TROP2 ADCs [15,76]. Payload resistance in tumor cells may upregulate drug efflux pumps (e.g., ABC transporters), reducing intracellular cytotoxicity [15,76]. Mutations in topoisomerase I or microtubule pathways may also confer resistance [73]. Altered internalization and trafficking within endosomes or lysosomes can impair ADC processing and payload release [73]. Micro-environmental factors, including hypoxia, stromal barriers, and tumor immune evasion, may limit ADC delivery or enhance survival signals [15].

In conclusion, patient selection and biomarker assessment are the cornerstones of ADC efficacy in NSCLC. Resistance is multifactorial, involving antigen loss, payload insensitivity, and tumor microenvironment factors. Future development of ADCs will require strategies to predict, monitor, and overcome resistance to maintain durable responses.

## 5. Future Directions of Non-HER2 ADCs

### 5.1. Next-Generation ADC Technologies

Emerging ADC platforms aim to enhance efficacy, minimize toxicity, and overcome resistance mechanisms observed in first- and second-generation constructs [77]. Key innovations include:

#### 5.1.1. Bispecific and Dual-Targeting ADCs

Bispecific and dual-targeting ADCs are engineered to bind two distinct antigens simultaneously or sequentially, potentially enhancing tumor selectivity and overcoming intra-tumoral heterogeneity that limits the efficacy of single-target ADCs [15,74,78]. These constructs may enhance internalization, broaden tumor-cell targeting, and reduce off-tumor toxicity by requiring co-expression of both antigens for optimal binding and payload delivery [15,78].

Preclinical studies have demonstrated that bispecific ADCs targeting combinations such as HER2 and TROP2, or other dual antigen pairs, exhibit enhanced cytotoxic activity across heterogeneous tumor models compared with monospecific ADCs, supporting their development for solid tumors [74,79,80]. Although most data to date come from non-lung cancer models, the principle of dual targeting is directly relevant to NSCLC, especially in tumors with heterogeneous antigen expression, where a single antigen may be insufficient for robust targeting [78]. Continued early-phase evaluation of these dual-antigen ADCs in NSCLC and other solid tumors will clarify their safety profile and translational potential [78].

#### 5.1.2. Conditionally Activated ADCs (Pro-ADCs)

Pro-ADCs or stimuli-responsive ADCs are engineered so that their linkers or payloads are selectively activated within the tumor microenvironment (e.g., tumor-associated proteases, acidic pH, hypoxia, or reactive oxygen species) to minimize premature systemic release and off-target toxicity [17]. These tumor-specific triggers allow the cytotoxic payload to remain inert in the circulation and to become activated only in the tumor bed [17]. This enhances tumor selectivity and potentially reduces adverse events in normal tissues. Preclinical models show that such designs can improve the therapeutic index compared with traditional ADCs by focusing cleavage and drug release where it is needed most [17].

Emerging strategies include hypoxia-sensitive linkers that are activated under low-oxygen tumor conditions and pH-responsive designs that exploit the acidic tumor milieu to trigger payload release [81]. While clinical experience with conditionally activated ADCs remains limited, preclinical evidence suggests a reduction in certain toxicities, such as off-target effects, and may ultimately lower the incidence of toxicities, including ILD and ocular effects, observed with classic ADCs [81].

#### 5.1.3. Novel Payloads

Novel ADC payloads encompass a broad array of highly potent cytotoxic mechanisms beyond traditional microtubule inhibitors, including DNA-alkylating agents, topoisomerase inhibitors, and emerging RNA polymerase inhibitors [17,82]. DNA-alkylating agents, such as PBD dimers and duocarmycins, form covalent adducts with DNA, leading to replication stress and apoptotic cell death; conversely, topoisomerase I inhibitors, such as DXd and SN-38, interfere with DNA replication and transcription, resulting in double-strand breaks and tumor cell apoptosis. These topoisomerase inhibitors are also membrane-permeable, enabling the therapeutic “bystander effect,” in which released payload reaches and kills adjacent antigen-low or antigen-negative tumor cells in heterogeneous tumors [17,82].

In addition to DNA-targeting mechanisms, novel payload classes, such as RNA polymerase inhibitors (e.g., α-amanitin derivatives), are being explored preclinically for incorporation into next-generation ADCs, thereby broadening the mechanistic diversity of payloads under investigation. Diversification of payload mechanisms aims not only to enhance cytotoxic potency but also to address antigen heterogeneity and improve clinical outcomes across diverse tumor types, including NSCLC, by optimizing both cell-intrinsic killing and bystander activity [17,82].

#### 5.1.4. Site-Specific Conjugation

Site-specific conjugation technologies have enabled the attachment of cytotoxic payloads at defined amino acid positions on the antibody, thereby ensuring a uniform drug-to-antibody ratio (DAR) and significantly reducing heterogeneity observed with conventional lysine- or cysteine-based conjugation methods [4,83,84]. This precise engineering improves ADC stability and pharmacokinetics. This is because homogeneous DAR species exhibit more predictable circulation half-lives, reduced premature de-conjugation, and enhanced bio-distribution compared with heterogeneous mixtures that vary widely in DAR and conjugation sites [4,83,84].

Multiple site-specific approaches, including engineered cysteine residues, non-natural amino acid incorporation, and enzyme-mediated coupling, have been shown to produce ADCs with enhanced therapeutic indices, greater in vivo efficacy, and improved safety profiles in preclinical studies [4,83,84]. By minimizing off-target release and aggregation, site-specific ADCs help decrease systemic toxicity while preserving or enhancing antitumor potency [4,83,84].

### 5.2. ADC Combinations and Sequencing Strategies

#### 5.2.1. Immunotherapy Combinations

ADCs have the potential to synergize with immune checkpoint inhibitors (ICIs) by inducing immunogenic cell death (ICD), releasing tumor antigens and danger-associated molecular patterns that enhance dendritic cell activation and T-cell priming [17,85]. Preclinical and early clinical evidence support this immunomodulatory effect, suggesting that ADC-induced tumor antigen release may sensitize tumors to PD-1/PD-L1 blockade and amplify antitumor T-cell responses [17,85].

Several ADC agents (such as Dato-DxD and sacituzumab govitecan) are being evaluated in combination with PD-1/PD-L1 inhibitors in ongoing trials across solid tumors, including NSCLC, to improve response rates and overcome immune resistance [2,17,85,86]. To this end, multiple clinical programs are investigating TROP2-DXd in combination with anti-PD-1/PD-L1 antibodies to determine whether these combinations can enhance efficacy beyond monotherapy in NSCLC and other solid tumors [17,85,87]. While results are preliminary, early safety and activity signals have been reported, supporting continued exploration of ADC-ICI combinations as a rational therapeutic strategy [17,85,86].

#### 5.2.2. TKI Sequencing

For patients with EGFR-mutated NSCLC, progression on TKIs such as osimertinib is nearly universal, driven by both on-target and bypass resistance mechanisms, and necessitates alternative therapeutic strategies beyond continued TKI monotherapy [57,75]. Retrospective reviews and expert-opinion articles highlight the rationale for incorporating ADCs, including those targeting HER3, MET, or other surface antigens, following TKI resistance, as they operate via distinct mechanisms that can bypass intracellular TKI resistance pathways [45,57,75,88,89]. Preclinical and early clinical data suggest that ADCs may retain activity in the setting of TKI resistance and, in some cases, show enhanced efficacy when combined with TKIs; for example, Teliso-V plus erlotinib demonstrated promising antitumor activity and tolerability in MET-overexpressing, osimertinib-resistant NSCLC [45,57,75,88,89].

Emerging evidence also supports the concept of combination therapy, integrating ADCs with TKIs or other targeted agents, to delay or overcome resistance before it fully emerges, such as dual EGFR/MET inhibition strategies and bi-specifics that simultaneously target resistance pathways [45,57,88]. The optimal sequencing of ADCs after TKIs (or in combination with them) remains an area of active investigation, underscoring the need for biomarker-guided approaches and prospective studies to inform best clinical practice.

#### 5.2.3. Chemotherapy and Radiotherapy Integration

Early-phase studies of ADCs combined with chemotherapy or radiotherapy indicate that such combinations are generally tolerable in heavily pretreated populations, though they often require careful dose modification and toxicity monitoring to manage overlapping adverse events [90]. However, combining ADCs with cytotoxic chemotherapy agents has been shown to increase the incidence of grade ≥3 toxicities, including neutropenia, fatigue, and stomatitis, due to overlapping cytotoxic effects of the payloads and chemotherapeutic drugs in several early-phase combination trials [90]. Likewise, radiotherapy combined with ADCs has demonstrated enhanced preclinical efficacy and potential radiosensitization, but also underscores the need for careful evaluation of normal tissue toxicity and sequencing to avoid additive damage, especially in lung tissue, where ILD risk may be amplified [91]. Collectively, these findings emphasize that optimization of ADC integration with chemo/radiotherapy requires prospective study designs with vigilant monitoring for myelosuppression, pulmonary toxicity (e.g., ILD), and other cumulative adverse events [90,91].

### 5.3. Biomarker-Driven Optimization

Future strategies in ADC development emphasize dynamic biomarker monitoring to refine patient selection and better predict responses, resistance, and optimal therapy sequencing. Non-invasive approaches include circulating tumor DNA (ctDNA) and circulating tumor cells (CTCs) [7,92,93]. This enables real-time monitoring of molecular changes during treatment, including shifts in target expression or the emergence of resistance mutations that may influence ADC efficacy [92,93]. For example, early decreases in ctDNA levels after therapy initiation have been correlated with improved clinical outcomes in NSCLC and may serve as early indicators of treatment response, potentially preceding radiographic changes [92,93]. Moreover, integrating ctDNA/CTC analyses with imaging and tissue biomarkers can help guide adaptive treatment decisions, such as switching ADCs or combining therapies before overt progression is detected [92,93]. As liquid biopsy technologies continue to improve in sensitivity and standardization, they hold promise for enhancing precision in ADC therapy across NSCLC and other solid tumors.

### 5.4. Overcoming Resistance

Typically, tumors resistant to one class of payload remain sensitive to another [94]. Employing alternative cytotoxic mechanisms in ADCs by payload switching can help circumvent resistance mediated by drug efflux pumps or enzymatic inactivation [94]. Drug efflux–mediated resistance is most commonly associated with topoisomerase I inhibitor payloads (e.g., DXd, SN-38), which are substrates of ATP-binding cassette (ABC) transporters such as ABCB1 (P-glycoprotein) and ABCG2, leading to reduced intracellular drug accumulation. In contrast, enzymatic or target-based resistance is more frequently observed with topoisomerase I inhibitors through alterations or mutations in the TOP1 enzyme, reducing drug binding and efficacy. Microtubule inhibitor payloads (e.g., MMAE, DM4) are also subject to resistance via efflux transporters, as well as through structural alterations in tubulin that impair drug binding. Therefore, switching between payload classes, such as from topoisomerase I inhibitors to microtubule inhibitors or vice versa, represents a rational strategy to overcome resistance and restore antitumor activity [94].

Dual-antigen or dual-payload ADCs (combining targets and/or mechanisms) are being developed preclinically to address tumor heterogeneity and reduce the impact of single-pathway resistance, demonstrating enhanced efficacy in models in which single-target ADCs fail [95]. In addition, integrating ADCs with rational combination therapies, including targeted agents, immunotherapies, or epigenetic modulators, may prevent or delay the emergence of escape pathways by simultaneously disrupting complementary survival signals and enhancing antitumor immunity [94]. Recent reviews highlight that bispecific or multi-target ADCs, payload diversification, and combination strategies represent cutting-edge approaches to overcoming conventional resistance mechanisms and improving the durability of response [94].

### 5.5. Expanding to Rare NSCLC Subtypes

ADCs may provide therapeutic options for patients with rare NSCLC histologies, including neuroendocrine-transformed NSCLC, MET-high adenocarcinomas, and EGFR-mutant disease after TKI resistance, where conventional therapies often yield limited benefit, while the benefit of ADCs in squamous NSCLC remains inconsistent across trials [96,97]. Early-phase studies and molecular profiling indicate that distinct NSCLC subpopulations defined by overexpression of ADC targets such as MET, CEACAM5, and TROP2 may be selectively susceptible to ADC therapy [13,96,97]. This provides a precision oncology approach beyond common driver mutations. Additionally, observational and early clinical data suggest that ADCs targeting novel antigens, including integrin β6, which is broadly expressed in subsets of NSCLC, show promising antitumor activity in heavily pretreated populations [98]. Such data underscores the feasibility of ADCs in uncommon tumor contexts and supports ongoing efforts to incorporate ADCs into treatment algorithms tailored to the molecular and histologic features of rare NSCLC subsets. This will potentially improve outcomes for which few effective therapies currently exist.

## 6. Conclusions

ADCs are among the most rapidly advancing therapeutic classes in thoracic oncology. Their emergence reflects a paradigm shift, leveraging the targeted delivery of potent cytotoxic agents to overcome traditional chemotherapy limitations while maintaining the precision of targeted therapy. Across NSCLC subtypes and molecular contexts, ADCs have demonstrated meaningful efficacy, even in heavily pretreated populations that have exhausted standard targeted therapy and immunotherapy options. They exhibit targeted cytotoxicity that can overcome established mechanisms of resistance. Agents such as HER3-DXd, Dato-DXd, Teliso-V, and Saci-tirumotecan demonstrate clinically meaningful activity across molecular subsets and histologies, filling therapeutic gaps after TKI failure and chemo-immunotherapy resistance.

The next era of ADC-based therapy in NSCLC will depend on refining multiple interconnected strategies. Rigorous biomarker standardization will be essential to ensure accurate patient selection and reproducibility across clinical trials. Sophisticated toxicity-mitigation frameworks, particularly for ILD, ocular toxicity, and cytopenias, will be required as more potent ADC platforms emerge. Integration with real-time molecular monitoring, such as ctDNA and dynamic antigen-expression profiling, will guide individualized dosing, assess early resistance, and optimize sequencing. Future clinical trial designs will increasingly incorporate ADCs into earlier lines of therapy, including perioperative and first-line metastatic settings. Parallel innovation in ADC engineering, including bispecific constructs, conditionally activated pro-ADCs, and next-generation payload platforms, will further expand therapeutic reach and minimize systemic toxicity. As these advances converge, ADCs are expected to become a foundational component of NSCLC management, offering durable responses and renewed therapeutic potential for patients with treatment-refractory disease.

## Figures and Tables

**Table 2:** ADCs Toxicity Profiles and Management in NSCLC.

Toxicity	Commonly Associated ADCs/Payloads	Typical Clinical Features	Management
Interstitial lung disease (ILD)/pneumonitis	DXd-based ADCs (e.g., Dato-DXd, HER3-DXd)	Cough, dyspnea, hypoxemia, ground-glass opacities on CT	Early detection, treatment interruption, corticosteroids; permanent discontinuation for grade ≥2
Myelosuppression	Topoisomerase I payloads (DXd, SN-38)	Neutropenia, anemia, thrombocytopenia	Dose modification, growth factor support, transfusion if needed
Peripheral neuropathy	Microtubule inhibitors (MMAE, DM4)	Paresthesia, numbness, sensory neuropathy	Dose interruption or reduction, symptomatic management
Ocular toxicity	DM4-based ADCs (e.g., tusamitamab ravtansine)	Keratitis, blurred vision, photophobia	Lubricating eye drops, ophthalmologic monitoring, dose modification
Gastrointestinal toxicity	Topoisomerase I inhibitors (DXd, SN-38)	Nausea, vomiting, diarrhea, stomatitis	Antiemetics, hydration, supportive care
Stomatitis/oral mucositis	DXd-based ADCs (e.g., Dato-DXd, HER3-DXd)	Oral ulcers, mucosal inflammation, pain	Prophylactic oral care, topical steroids, dose modification
Fatigue	Multiple ADC classes	Generalized weakness, decreased performance status	Supportive care, dose modification if severe

Table is adapted from pooled DXd ILD analyses, T-DXd safety reports, and ADC toxicity reviews [24,25,30,31,32,33,34,35,36]. ADC: antibody–drug conjugate. CT: computed tomography. DM4: maytansinoid microtubule inhibitor. DXd: deruxtecan. ILD: interstitial lung disease. MMAE: monomethyl auristatin E. NSCLC: non-small cell lung cancer. SN-38: 7-ethyl-10-hydroxycamptothecin.

**Table 3:** Clinical Trial Efficacy Summary of Major Non-HER2 ADCs in NSCLC.

ADC	Target	Trial	Phase	Population	Biomarker Selection	ORR	mPFS	mOS	mDoR	Key Safety Findings
Datopotamab deruxtecan (Dato-DXd)	TROP2	TROPION-Lung01	III	Previously treated advanced/metastatic NSCLC after platinum-based chemotherapy ± immunotherapy	Not prospectively selected by TROP2 expression in the pivotal trial	26.4%	4.4 months	12.9 months	-	Grade ≥ 3 TRAEs 25.6%; adjudicated drug-related ILD/pneumonitis 8.8%
		TROPION-Lung01 non-squamous subgroup		non-squamous subgroup	Histology-based subgroup	-	5.5 months	14.6 months	-	Benefit favored Dato-DXd over docetaxel; ILD remains relevant
		TROPION-Lung01 squamous subgroup		squamous subgroup	Histology-based subgroup	-	2.8 months	7.6 months	-	No efficacy advantage over docetaxel in this subgroup
		TROPION-Lung05	II	Advanced/metastatic NSCLC with actionable genomic alterations after prior targeted therapy and platinum-based chemotherapy	Molecularly selected population with actionable genomic alterations, including EGFR-mutated tumors	43.6%	-	-	~6.5 months	Safety profile generally manageable; common TRAEs included stomatitis, nausea, and fatigue; adjudicated ILD occurred in a small proportion
Sacituzumab govitecan (SG)	TROP2	Single-arm multicenter trial	I/II	Heavily pretreated metastatic NSCLC	No clear predictive role for Trop-2 despite high expression in assessable archival samples	~17%	5.2 months	9.5 months	-	Grade ≥ 3 TEAEs: neutropenia 28%, diarrhea 7%, nausea 7%, fatigue 6%, febrile neutropenia 4%
		EVOKE-01	III	Previously treated advanced/metastatic NSCLC	Not biomarker selected	-	-	No OS benefit vs. docetaxel	-	Safety profile generally consistent with known SN-38-based ADC toxicities; confirmatory benefit in the overall population was not demonstrated
Sacituzumab tirumotecan (MK-28701)	TROP2	OptiTROP-Lung04	III	EGFR-mutated advanced/metastatic NSCLC after progression on EGFR TKIs	Molecularly selected (EGFR-mutant population)	-	8.3 months	18-month OS rate: 65.8% (vs. 48% chemotherapy)	-	Manageable safety profile; hematologic and gastrointestinal toxicities consistent with topoisomerase I-based ADCs
Patritumab deruxtecan (HER3-DXd)	HER3	HERTHENA-Lung01	II	Advanced EGFR-mutated NSCLC previously treated with EGFR TKI and platinum-based chemotherapy	EGFR-mutant NSCLC; activity observed across HER3 expression levels and resistance mechanisms	29.8%	5.5 months	11.9 months	6.4 months	Adjudicated ILD 5.3%; nausea, stomatitis, fatigue, thrombocytopenia, neutropenia
Izalontamab brengitecan (BL-B01D1)	EGFR/HER3	First-in-human study	I	Advanced solid tumors including EGFR-mutant NSCLC after progression on third-generation EGFR TKIs	Not biomarker-restricted; activity observed across resistance mechanisms	~44–50%	-	-	~6–8 months (immature)	Manageable safety profile; common toxicities included neutropenia, nausea, and diarrhea; safety consistent with topoisomerase I-based ADCs
Telisotuzumab vedotin (Teliso-V)	MET	LUMINOSITY	II	Locally advanced/metastatic non-squamous EGFR wild-type c-Met-overexpressing NSCLC with ≤2 prior lines	c-Met overexpression by IHC	28.6% overall	5.7 months	14.5 months	8.3 months	Peripheral sensory neuropathy, peripheral edema, fatigue; grade ≥ 3 neuropathy 7%
		LUMINOSITY MET-high subgroup		MET-high non-squamous NSCLC	MET-high by IHC	34.6%	5.5 months	14.6 months	9.0 months	Toxicity profile consistent with MMAE-based ADCs
		LUMINOSITY MET-intermediate subgroup		MET-intermediate non-squamous NSCLC	MET-intermediate by IHC	22.9%	6.0 months	14.2 months	7.2 months	Toxicity profile consistent with MMAE-based ADCs
Tusamitamab ravtansine (SAR408701)	CEACAM5	Dose-expansion study	I/II	Non-squamous NSCLC with high or moderate CEACAM5 expression	CEACAM5 expression by IHC	20.3% (high), 7.1% (moderate)	-	-	6.7 months (high)	TRAEs in 78.3%; corneal adverse events 38.0%, mostly grade 1/2 and reversible
		CARMEN-LC03	III	Biomarker-selected population of previously treated advanced non-squamous NSCLC	CEACAM5-positive IHC, typically moderate-to-high expression	-	Did not improve vs. docetaxel	No OS benefit	-	Ocular (corneal) toxicity remained a characteristic adverse event.

Table is derived from the primary trial publications and key development reports for each ADC [10,11,20,22,23,25,27,37,38,39,40,41,42,43,44,45]. ADC: antibody–drug conjugate. CEACAM5: carcinoembryonic antigen-related cell adhesion molecule 5. DXd: deruxtecan. EGFR: epidermal growth factor receptor. HER3: human epidermal growth factor receptor 3. IHC: immunohistochemistry. ILD: interstitial lung disease. mDoR: median duration of response. MET: mesenchymal–epithelial transition receptor. mOS: median overall survival. mPFS: median progression-free survival. MMAE: monomethyl auristatin E. NSCLC: non-small cell lung cancer. ORR: objective response rate. SG: sacituzumab govitecan. TKI: tyrosine kinase inhibitor. TRAEs: treatment-related adverse events. TEAEs: treatment-emergent adverse events.

## Data Availability

No new data were created or analyzed in this study. Data sharing is not applicable to this article.
